# Improving engineering properties of laterite soil using eco-friendly biopolymers: a study on strength and compressibility

**DOI:** 10.1038/s41598-026-43269-2

**Published:** 2026-03-26

**Authors:** Ahmad M. Ebid, Shailendra Banne, Snehal U. Bobade, Raviraj Sorate

**Affiliations:** 1 Future University in Egypt, Cairo, Egypt; 2 Department of Civil Engineering, Pimpri Chinchwad College of Engineering, Pune, 411044 India; 3 Department of Civil Engineering, COEP Technological University, Pune, 411005 India; 4 Department of Civil Engineering, Anantrao Pawar College of Engineering and Research, Pune, 411009 India

**Keywords:** Laterite soil, Biopolymers, Xanthan gum, Guar gum, UCS, CBR, Compressibility, XRD analysis, Engineering, Environmental sciences, Materials science

## Abstract

In recent years, there has been a growing focus on sustainable and eco-friendly alternatives to conventional chemical stabilizers for improving problematic soils. This study investigates the stabilization of laterite soil using eco-friendly biopolymers xanthan gum (XG) and guar gum (GG) to enhance its engineering properties. Soil samples treated with 1% to 4% biopolymer dosages were tested after curing periods ranging from 3 to 28 days. Laboratory evaluations included Unconfined Compressive Strength (UCS), permeability, and consolidation tests, along with microstructural analyses using XRD. The results demonstrated significant improvements in strength, reduced permeability, and enhanced microstructural integrity, confirming the potential of XG and GG as sustainable soil stabilizers. Results reveal that 3% XG and 2% GG are the optimum concentrations, yielding maximum UCS improvements of 321.40% and 241.05% after 28 days, respectively. Permeability reduced significantly by 93.12% for 3% XG and 96.96% for 2% GG, while void ratio decreased from 0.807 to 0.4803 and 0.454, indicating enhanced consolidation. Microstructural analysis confirmed formation of cementitious products and dense matrices contributing to strength gains. The findings of this study provide practical insights into using biopolymers as green stabilizing agents to enhance the strength of laterite soils for sustainable geotechnical applications.

## Introduction

Laterite soils are commonly found in tropical regions, including vast parts of India, and are frequently used in construction activities such as road subgrades and embankments^1^. However, their engineering behaviour particularly strength, permeability, and compressibility often limits their performance in load-bearing applications. To enhance their suitability for such uses, soil stabilization becomes necessary. Stabilization indicates a technique employed to improve the strength characteristics of soil through the incorporation of additional material into the present soil^2^. Soil improvement is an important long-term solution that has many environmental, economic, and social benefits^3,4^. Stabilizing laterite soil is the subject of many studies. Researchers have looked into what lime and cement do to laterite soil^5–7^. Carbon and energy burden Cement-based soil stabilization is widely practiced, yet it carries substantial environmental burdens. Portland cement manufacture accounts for roughly 7–8% of global anthropogenic CO^2^ emissions, driven by limestone calcination and the high thermal energy demand of clinker production^8^. Beyond process emissions, cement production entails intensive extraction of non-renewable raw materials and significant upstream energy use for quarrying, grinding, and transport^9^. In stabilization practice, higher cement dosages often required in iron- and aluminum-rich laterite soils can further amplify embodied emissions per unit mechanical gain^10^. Recent studies show that engineering sediment waste can serve as a sustainable subgrade material when activated with GGBS, electrolytic manganese residue, and limited cement, reducing the environmental impact of cement-based stabilization^11^. Increased lime concentration to 12% resulted in optimum strength, 86.2% compaction rate, and 112.44% field CBR value for road bases in laterite soil^12^. Several researchers have explored the enhancement of laterite soil properties using various additives such as fly ash^13^, bagasse ash^14^, and rice husk ash (RHA)^15^. A physical model study on laterite road bases in Halmahera demonstrated a notable decrease in deformation with the application of 10% lime content^16^. Another investigation examined the time-dependent reactivity of laterite soil with two less conventional additives, TX-85 and SH-85, through macro- and microstructural analysis^17^. Other materials used for improving soil characteristics include tire-derived aggregates, 3% microsilica^18^, groundnut shell ash^19^, calcium chloride (CaCl₂)^20^, polymer-based soil stabilizer SS299^21^, agricultural residues^22^, cement combined with quarry dust^23^, lignin^24^, and cellulose^25^. In 2015, the United Nations introduced the Sustainable Development Goals (SDGs) under the 2030 Agenda, comprising 17 global objectives aimed at eradicating poverty, protecting the planet, and promoting peace and prosperity for all by the year 2030^26^. Biopolymer stabilization supports Sustainable Development Goals by promoting eco-friendly ground improvement techniques that reduce environmental impact and enhance infrastructure resilience. In recent years, biopolymers have emerged as promising eco-friendly alternatives, offering significant geotechnical improvements with minimum environmental impact^27–31^. XG and GG are two biopolymers that have acquired popularity due to their binding qualities, water retention capacities, and biodegradability.

### Studies on Xanthan gum (XG) biopolymer

Studies have demonstrated that XG, at optimum contents of 1–1.5%, significantly improves the unconfined compressive strength and reduces the compressibility of montmorillonite and kaolinite clays over a 28-day curing period, with microstructural analyses (FESEM and BET) confirming the formation of cementitious bonds that weld soil particles together and fill pore spaces, thereby enhancing the overall soil structure^32^. Experimental results from road construction in Sri Lanka showed that soils treated with XG biopolymer exhibited significantly higher unconfined compressive strength and ductility than those stabilized with cement-ash binders, confirming its effectiveness as a sustainable and high-performance stabilizer for road shoulders and subbases^33^. Studies have shown that XG, when mixed with sand, clay, and silty sand, significantly enhances unconfined compressive strength, shear strength, and triaxial parameters, demonstrating its effectiveness as an environmentally friendly alternative to cement for improving weak soils and supporting its potential in sustainable construction practices^34^. This study evaluates XG as eco-friendly stabilizer for collapsible soils. Laboratory tests showed that biopolymer addition reduced dry density and permeability, while improving mechanical properties based on curing time and dosage^35^. XG added in varying amounts (0–1.0%) to expansive soil improves strength and durability while reducing swelling and hydraulic conductivity. Though plasticity initially increases, it drops beyond 0.5% XG, and microstructural changes confirm gel-like formations enhancing soil stability^36^. XG improved expansive soil properties by increasing UCS fourfold and reducing compressibility by 65% at 1% dosage after 28 days. It also enhanced shear strength, with SEM confirming gel formation and pore filling, making it an eco-friendly stabilizer^37^. XG significantly improves the strength and slope stability of laterite soil under both normal and submerged conditions. In the Lote Parshuram Ghat region, XG treatment (1–5%) increased soil cohesiveness by up to 378.64% and dry density by 28.36%. Slope stability analysis using Plaxis LE 2D showed a 57.25% rise in factor of safety under submerged conditions, with 2.7% XG found optimal for maximum stability^38^. XG effectively improves expansive subgrade soil, with 1.5% content found optimal for strength and stiffness gains over aging periods up to 365 days. Key properties like UCS, elastic and resilient modulus, and CBR increased by 5–10 times, while microstructural analysis confirmed strength gain due to XG-hydrogel bridging^39^. XG enhances the strength of lateritic soil by increasing UCS and shear parameters with higher dosages (0.5–1.5%) and longer curing times (3–14 days). FESEM analysis confirmed the formation of cementitious gels that fill soil pores, creating a denser and more continuous structure^40^. XG significantly improves soil strength and reduces hydraulic conductivity by forming hydrogel networks and promoting soil-biopolymer interactions. Even a small addition of 0.25% XG reduces permeability by nearly 1000 times, while strength and stiffness also increase, making it a highly effective stabilizer for geotechnical applications^41^. XG biopolymer, mixed with sand (0.075–1.0 mm) in varying ratios (0–1.5%), effectively reduces permeability and enhances compressibility, strength, and stiffness. Lab tests confirm its potential to improve soil performance for sustainable ground improvement^42^. XG considerably increased the strength of kaolin clay, with UCS increasing by 5.23 and 8.53 times, respectively, after 90 days of curing. The study also observed long-term strength retention over 3 years, highlighting biopolymers’ potential for sustainable soil improvement despite their organic nature^43^. XG improves soil strength by forming matrices that bind soil particles, especially in well-graded fine soils. Its effectiveness depends on soil type, moisture content, gum concentration, and mixing method, making it a promising eco-friendly alternative to conventional stabilizers^44^. After 28 days, XG increases tropical organic peat shear strength sixfold. Microstructural investigations showed that cementitious materials densify soil structure, supporting its usage as a sustainable stabilizer alternative^45^. In the Konkan region, where laterite soil covers about 70.7% area and weakens upon water exposure, XG was tested as a sustainable stabilizer. Using 1–3% XG increased cohesiveness by 35% and reduced internal friction angle by 20%^46^.

### Studies on Guar gum (GG) biopolymer

Laterite soil stabilized with GG exhibited a notable enhancement in strength, with unsoaked and soaked CBR values rising by 148% and 192.36%, respectively. Cohesion and internal friction angle increased 93.33% and 31.52%. GG is a sustainable, eco-friendly soil stabilizing alternative with long-term benefits, but pavement costs 1.5–1.7 times more^47^. GG biopolymer effectively reduces swell–shrink behavior in expansive clays, helping to mitigate shallow slope failures in embankments. Experimental investigations and slope stability analysis revealed that GG enhances the strength of soil and raises the safety factor, presenting an eco-friendly substitute to conventional stabilizing agents for ensuring long-term slope stability^48^. GG, a natural biopolymer, effectively enhances the strength of expansive soils, especially with increased curing periods. Tested across concentrations from 0.5% to 3%, it improved unconfined compressive strength and CBR values, making it a sustainable and eco-friendly alternative for soil stabilization in pavement design^49^. GG -treated soil shows improved strength and reduced compressibility due to hydrogel formation, with strength increasing up to 131% after 90 days. It offers a sustainable stabilization method with minimal sensitivity to water content and compaction energy^50^. Kuttanad clay, known for low strength and high compressibility, was stabilized using GG. GG led to a 59.5% UCS gain and 17.6% drop in OMC, highlighting their effectiveness in improving soil properties^51^. GG (0.25–2%) significantly increased shear strength and drastically reduced permeability in both cohesive and cohesionless soils over 1–10 weeks of curing^52^.

The novelty of this study lies in evaluating the potential of eco-friendly biopolymers, XG and GG, to improve the engineering properties of laterite soil as a sustainable alternative to conventional chemical stabilizers. The research investigates the effects of varying biopolymer dosages on the strength and compressibility characteristics of the soil through unconfined compressive strength, direct shear, and consolidation tests. The outcomes provide valuable insights into the biopolymer–soil interaction mechanisms for sustainable ground improvement applications.

### Objectives

This study examines at how varying dosages of XG and GG affect the unconfined compressive strength (UCS), permeability, and compressibility of laterite soil. The investigation includes several curing times to assess the time-dependent increase in soil parameters. In addition, microstructural analysis using X-ray diffraction (XRD) to study the internal alterations in the soil matrix caused by biopolymer treatment. The research aims to establish an optimum biopolymer dosage for maximum performance enhancement and to promote sustainable practices in geotechnical engineering by integrating bio-based materials in soil stabilization.

## Methodology and experimental plan

### Methodology

**Fig. 1:**
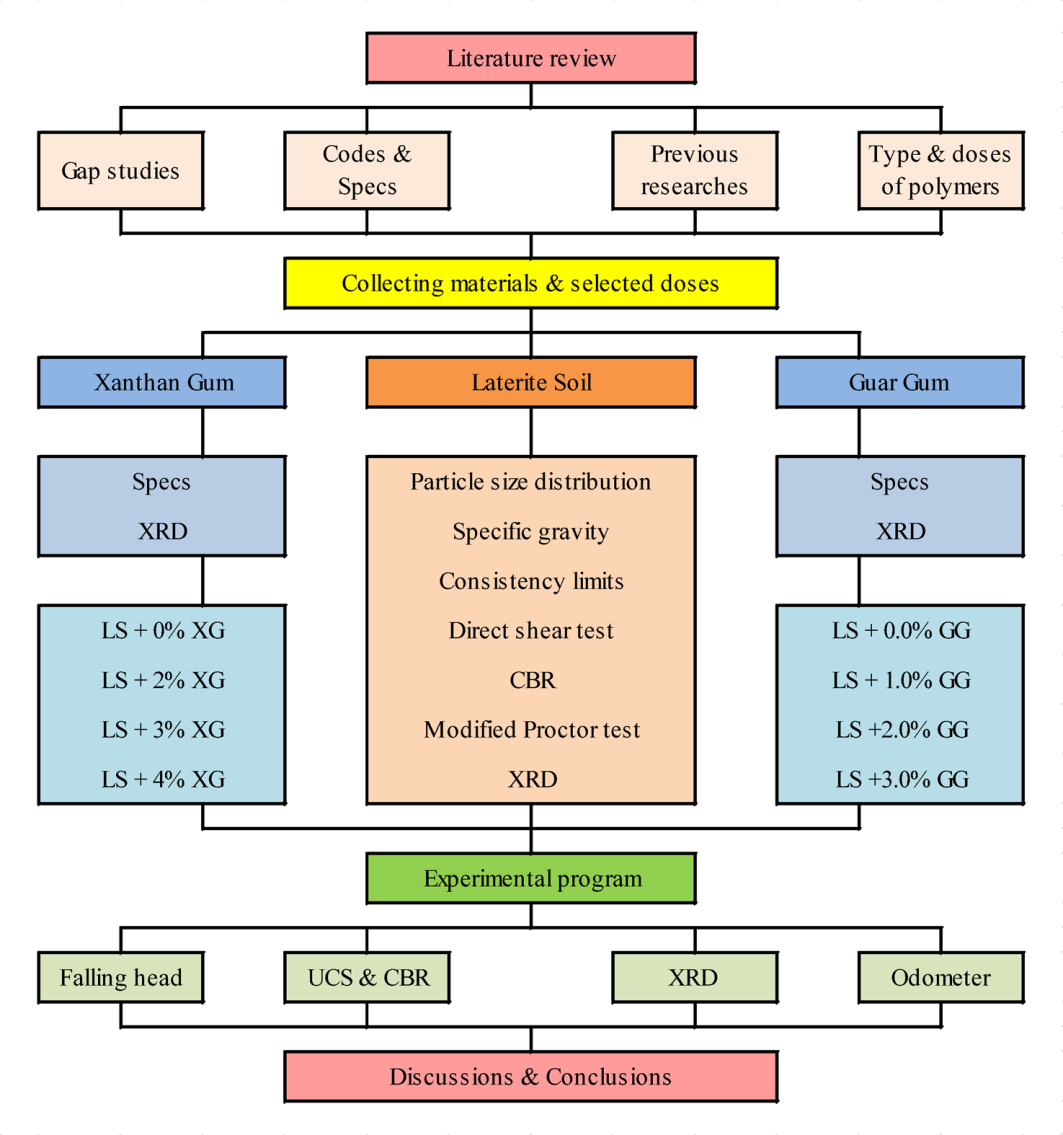
Methodology adopted.

The method used in this study is based on testing how different types and amounts of biopolymers affect the properties of laterite soil. For this, six tests were done on mixtures of laterite soil with three different doses of each biopolymer, along with a control test using only laterite soil. The tests included UCS, CBR, falling head permeability, consolidation (odometer), and XRD. The results were compared and discussed to understand how each biopolymer mixture changes the soil properties. Figure 1 show the methodology adopted in present study.

### Experimental plan

Experiments were in a room with a temperature of roughly 25 to 270 C and a relative humidity of 54 to 56% throughout the tests. The engineering and geotechnical characteristics of the mixture have been analyzed and figured out in line with the criteria laid out by the Indian Standards (IS). The comprehensive layout of the experimental program is summarized in Table 1.

**Table 1:** Testing program.

Type of biopolymer	Dosage (% by dry weight of soil)	Test type	Curing period (days)	No. of replicates
Xanthan gum (XG)	0.00 (Control)	UCS	0, 3, 7, 14, 28	3
	2	UCS	0, 3, 7, 14, 28	3
	3	UCS	0, 3, 7, 14, 28	3
	4	UCS	0, 3, 7, 14, 28	3
	0.00 (Control)	CBR, Permeability, Consolidation	0	3
	2	CBR, Permeability, Consolidation	0	3
	3	CBR, Permeability, Consolidation	0	3
	4	CBR, Permeability, Consolidation	0	3
Guar gum (GG)	0.00 (Control)	UCS	0, 3, 7, 14, 28	3
	1	UCS	0, 3, 7, 14, 28	3
	2	UCS	0, 3, 7, 14, 28	3
	3	UCS	0, 3, 7, 14, 28	3
	0.00 (Control)	CBR, Permeability, Consolidation	0	3
	1	CBR, Permeability, Consolidation	0	3
	2	CBR, Permeability, Consolidation	0	3
	3	CBR, Permeability, Consolidation	0	3

## Materials

### Laterite soil

**Fig. 2:**
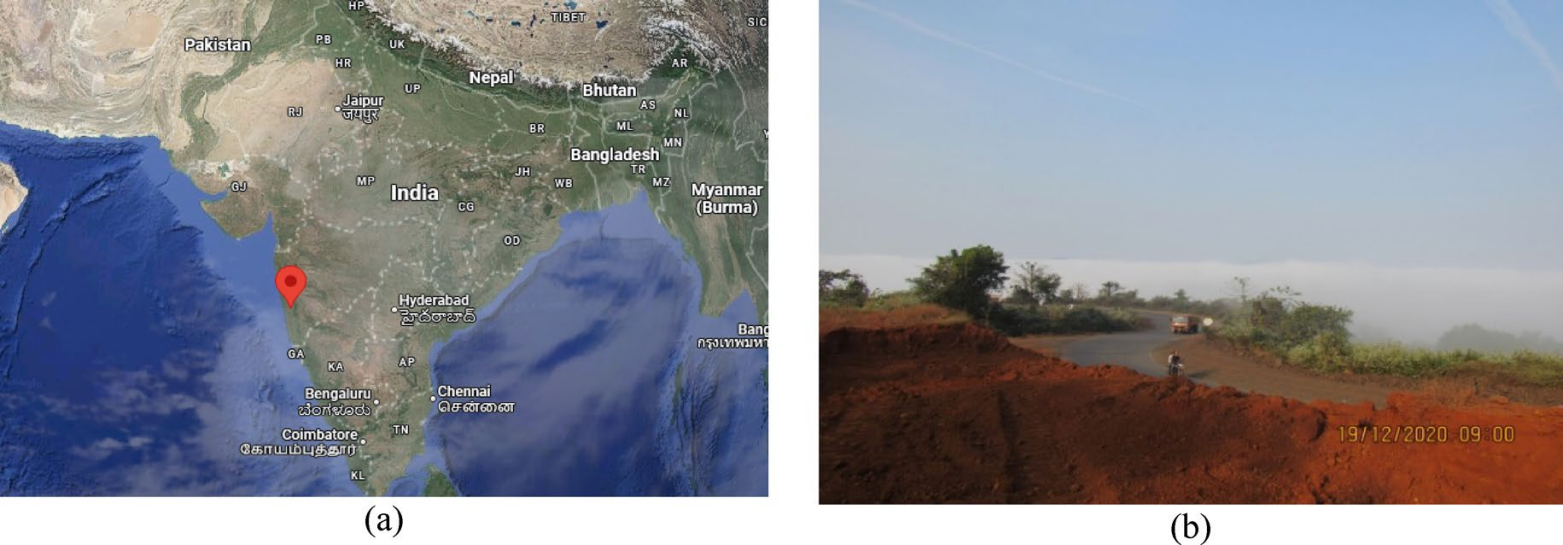
(**a**) Map of laterite soil sample collection (**b**) Soil collection site (17°33’28.0"N, 73°29’53.0"E).

**Fig. 3:**
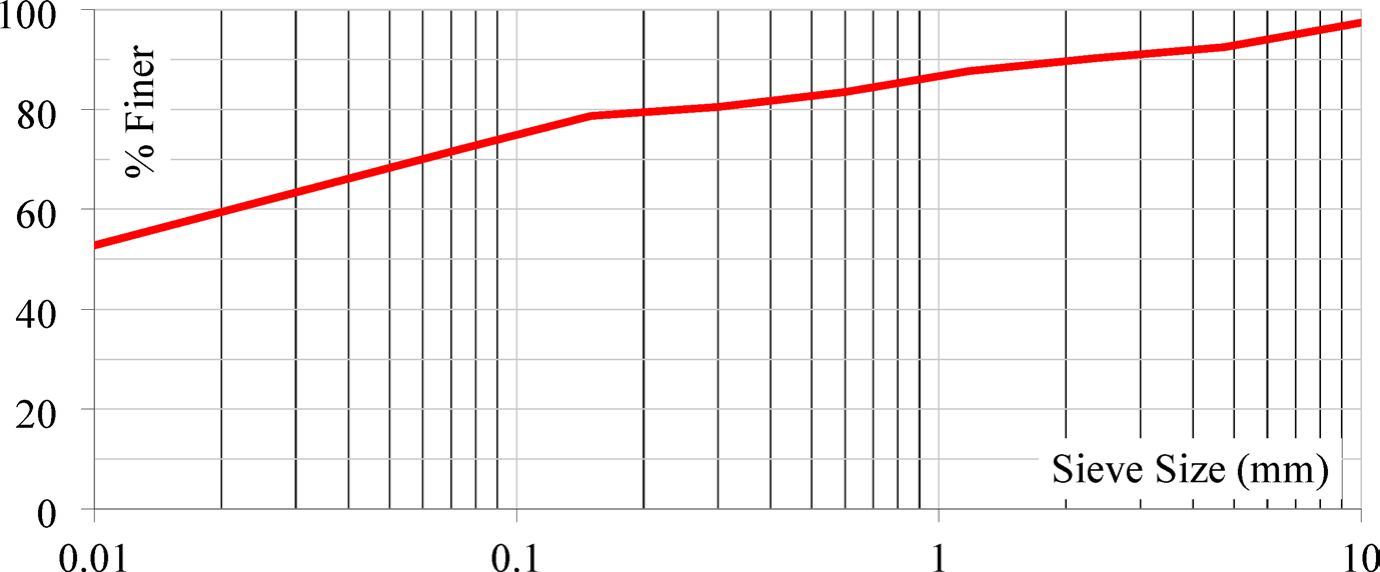
Particle size distribution curve of laterite soil.

**Fig. 4:**
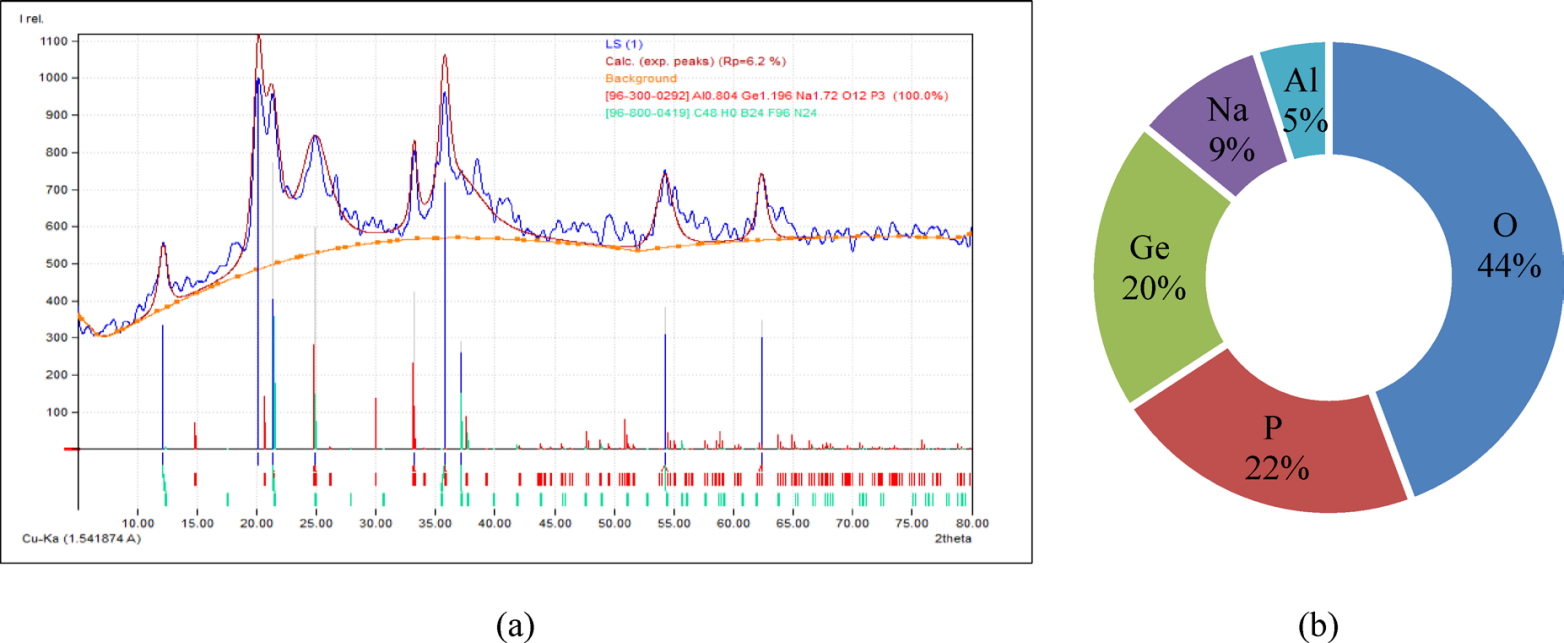
(**a**) XRD pattern (**b**) Elemental composition analysis of virgin laterite soil.

Laterite soil is reddish or yellowish and poor in nitrogen and manganese oxides. High temperatures, heavy rainfall, and alternating wet and dry circumstances cause leaching of iron and aluminium oxides. For this investigation, laterite soil samples were collected from Lote Parshuram Ghat, Chiplun, Maharashtra (17°33’28.0"N, 73°29’53.0"E). Figure 2a illustrates the location of soil sampling, while Fig. 2b presents the actual site of soil collection. Figure 3 show the particle size distribution curve, while Table 2 summarizes the geotechnical parameters of the collected virgin laterite soil. Figure 4a presents the XRD pattern of the virgin laterite soil, and Fig. 4b shows its elemental composition.

**Table 2:** Typical properties of field-collected laterite soil.

Properties	Value	Reference
Particle size distribution		
Sand %	32–37	IS: 2720 (Part-4): 1985
Silt %	20–24	
Clay %	48–52	
Specific gravity (G)	2.56–2.72	IS 2720 (Part-3) 1980
Soil classification	MH	
Consistency limits		
LL (%)	52–60	IS 2720 (Part-5) 1985
PL (%)	30–36	
PI (%)	22–24	
SL (%)	18–24	
CBR value (%)		
Soaked	7.07	IS 2720 (Part-16) 1987
Unsoaked	8.37	
Direct shear test		
C (kg/cm^2^)	0.15–0.17	IS 2720 (Part-15) 1986
φ (Degree)	15.10–17.20	
Modified proctor test		
MDD (gm/cc)	1.45–1.55	IS 2720 (Part-8) 1980
OMC (%)	13.10–17.40	

### Xanthan gum (XG) biopolymer

**Fig. 5:**
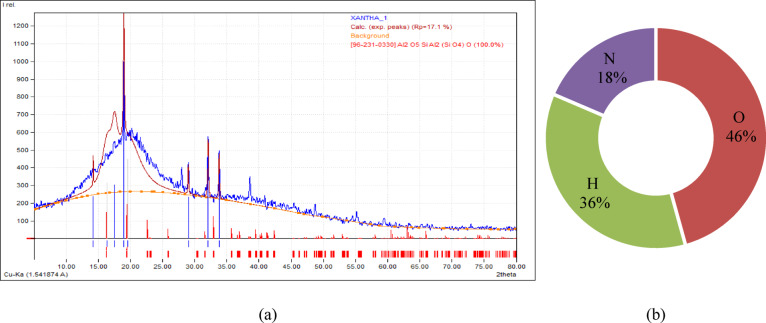
(**a**) XRD pattern (**b**) Elemental composition analysis of XG biopolymer.

Fermentation of Xanthomonas campestr is bacteria produces XG. Its high molecular weight and water retention make it viscous and gel-forming at low concentrations. Xanthan gum’s high oxygen, hydrogen, and nitrogen concentration makes it appropriate for soil stabilization. Because of its water retention, cohesion-enhancing qualities, shear strength enhancement, erosion control, and biodegradability, it helps stabilize soil and prevent erosion. Figure 5a exhibit XG biopolymer’s XRD results, which reveal its elemental composition in Fig. 5b.

### Guar gum (GG) biopolymer

**Fig. 6:**
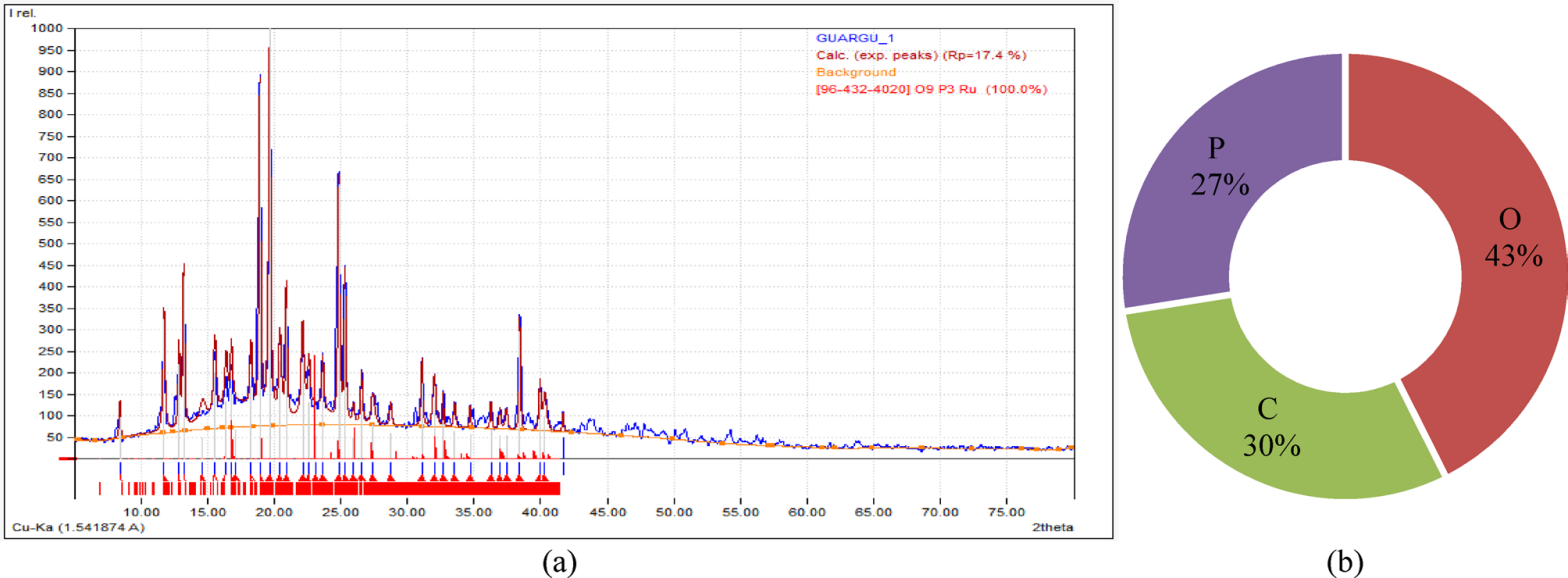
(**a**) XRD pattern (**b**) Elemental composition analysis of GG biopolymer.

Guar gum is a biopolymer formed from the endosperm of the seeds of the guar plant, Cyamopsis tetragonoloba. Because of its distinctive properties, it is a natural thickening and stabilizing agent widely employed in various industries. Guar gum’s propensity to generate highly viscous solutions when mixed with water is one of its key features. XRD investigation results show in Fig. 6a and its elemental composition in Fig. 6b. Table 3 shows the specifications of XG and GG biopolymer.

**Table 3:** Specifications of biopolymers.

Item	Specifications of biopolymers	
	Xanthan gum	Guar gum
Physical state	White to off-white	White to off-white
pH	6 to 8	5.5 to 7.5
Viscosity (1% solution in 1% KCl)	1400–1800 cps	5010–5300 cps
Moisture content	Max 12%	Max 12%
Particle size	#80–100% pass #200 − 92% pass	#200 − 97% pass
Ash content	Max 13%	Max 13%

### Mixing method

**Fig. 7:**
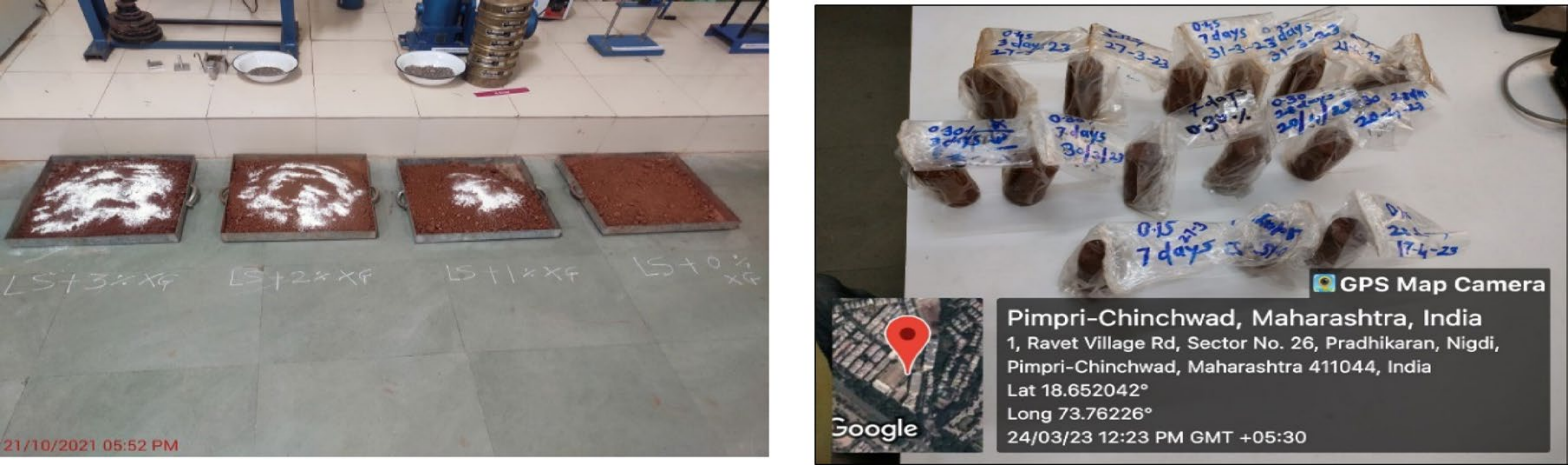
(**a**) LS + XG (Dry Mixing) (**b**) Airtight UCS specimens.

Xanthan gum (1% to 4%) and guar gum (1%` to 3%) were mixed with the laterite soil in varying proportions of by dry weight of soil. The selected dosage range was based on previous research findings and preliminary laboratory trials, which indicated that biopolymer contents within this range provide noticeable improvement in the strength and compressibility characteristics of soil. To ensure reliable performance, slightly higher dosages than the generally suggested concentrations were adopted. All biopolymer-treated and untreated samples were maintained in a controlled laboratory environment at a temperature of 25 ± 2 °C and high humidity conditions to prevent moisture loss. Each compacted specimen was sealed in airtight polyethylene bags immediately after preparation and stored for curing periods of 0, 3, 7, 14, and 28 days before testing. Figure 7a shows laterite soil mixed with XG biopolymer by dry mixing and Fig.7b depicts the airtight UCS samples.

## Experimental program results

### UCS test

#### Effect of XG

**Fig. 8:**
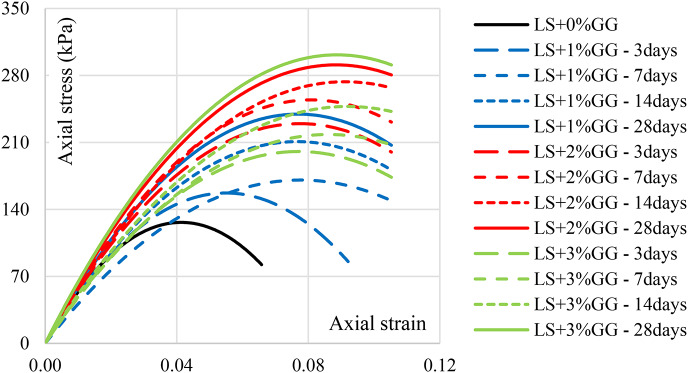
Stress-strain curves of untreated and XG-treated laterite soil.

The unconfined compressive strength (UCS) of the laterite soil demonstrated a significant relationship with the XG content. A consistent improvement in UCS was observed with increasing curing duration, particularly at the optimum dosage of 3%. For untreated laterite soil, 2% XG treated laterite soil, 3% XG treated laterite soil, and 4% XG treated laterite soil, the UCS at 0 days (shortly after soil sample preparation) is 110.50 kPa. This value increases by 25.15%, 37.50%, and 25.15% for every 1% XG content. For 3% XG content and various curing times (0, 3, 7, 14, and 28 days), the corresponding UCS values are 151.94, 332.28, 360.39, 415.84, and 443.56 kPa. Figure 8 illustrates the stress-strain behavior of both untreated and XG-treated soil samples over different curing intervals.

**Fig. 9:**
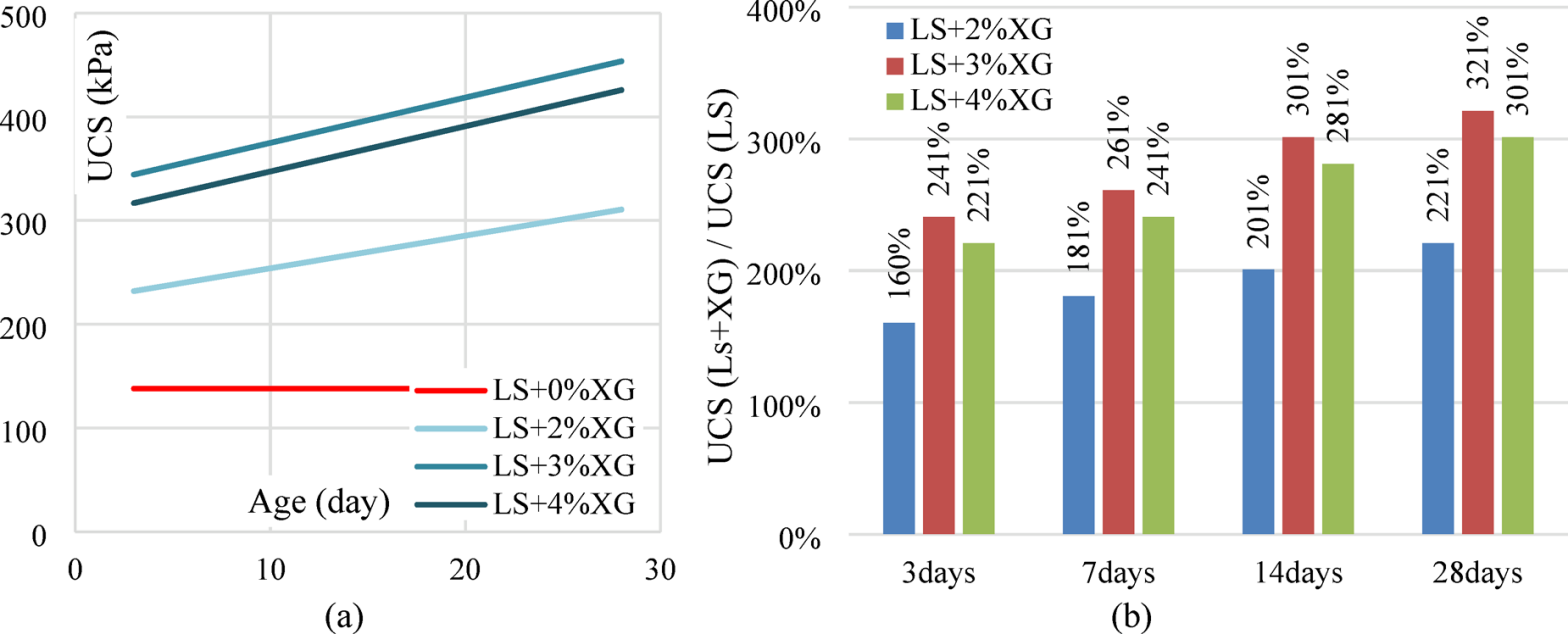
(**a**) Variation of UCS values with XG at various curing ages (**b**) % increase in UCS value with XG at various curing times.

Only about 3% of XG material seems to have any real value. Cation bridging and hydrogen bonding can prevent vacuum zones in the XG soil matrix, creating robust, mechanically superior soil matrices. The compressive strength of XG is enhanced by hydrogen bonding and interactions with soil particles. UCS peaked at 28 days and 3% XG. On the 28th day, UCS increased 221%, 321%, and 301% for 2%, 3%, and 4% XG. The variation in UCS values with different XG contents and curing durations is illustrated in Fig. 9a, while the % increase in UCS with respect to curing time is presented in Fig. 9b.

#### Effect of GG

**Fig. 10:**
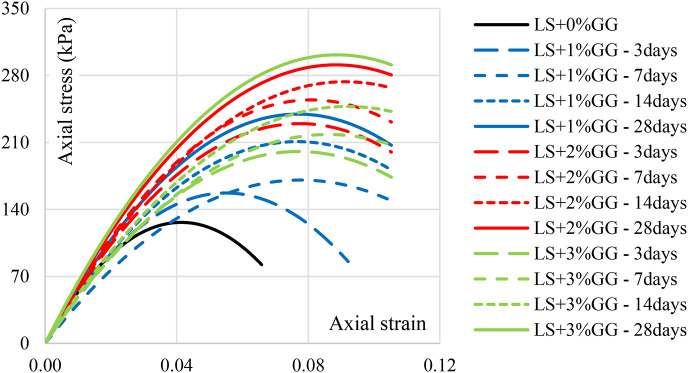
Stress-strain curves of untreated and GG-treated laterite soil.

The addition of GG improves the compressive strength of laterite soil, with strength increasing at higher GG dosages. At 28 days of curing, the strength further enhances due to improved particle bonding and continued interaction between the soil and biopolymer, indicating GG’s effectiveness as an eco-friendly stabilizer for laterite soils. Figure 10 illustrates the stress–strain behavior of laterite soil treated with varying GG contents at different curing periods. The results show that axial stress increases with both GG dosage and curing duration, indicating improved bonding and stiffness of the treated soil. The 3% GG mix at 28 days exhibits the highest peak strength, confirming the progressive enhancement of mechanical properties with curing time.

**Fig. 11:**
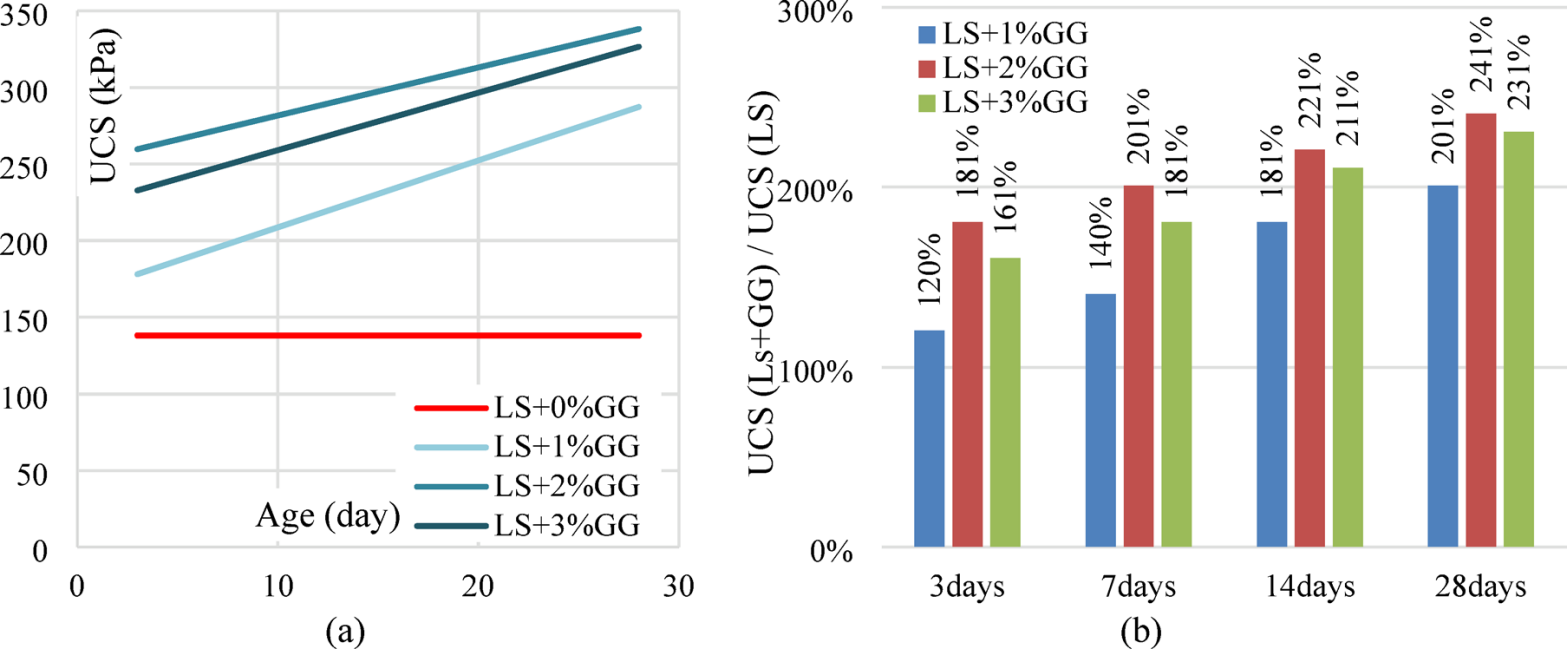
(**a**) Variation of UCS values with GG at various curing ages. (**b**) % increase in UCS value with GG at various curing times.

The UCS for 0 day is 110.50 for untreated laterite soil, 1%, 2%, and 3% GG treated laterite soil, respectively, representing a 25.29%, 50.35%, and 37.98% increase for each 1% GG content. Figure 11a shows, for 28 days, the UCS is 166.14, 249.5, 277.22, 304.94, and 332.67 kPa for 2% GG content at different curing periods like 0, 3, 7, 14 and 28 days respectively. The UCS increases with an increase in GG Content as the curing period lengthens. This study determined that a concentration of 2% GG was optimum. UCS value decreases with increasing GG content after 2% GG, shows in Fig. 11b. As with other curing times, expanding the GG content strengthens the laterite soil. The soil treated with 2% GG had a compressive strength increase of about 201.05%, going from 110.50 kPa before curing to 332.67 kPa after 28 days. This suggests that raising the GG concentration significantly improves the soil’s shear strength. Numerous hydroxyl groups joining together to create hydrogen bonds caused the rise in strength. The maximum UCS was recorded at 28 days and 2% GG concentration. The percentage increase in UCS on the 28th day was 201.05%, 241% and 231% for 1% GG, 2% GG and 3% GG respectively.

### Direct shear test results

Shear resistance of laterite soil and biopolymer blends can be assessed through a direct shear box examination. The soil blend’s shear resistance represents its capacity to withstand external forces. Four standard stress levels (0.5, 1, 1.5, and 2 kg/cm^2^) were used in the experiments. Shearing was performed at a constant rate of 0.2 mm/min. When biopolymers such as XG, GG are added to laterite soil, they can enhance cohesion. In the case of XG, the optimum dose is 3% of the total soil mass. The optimum amount for GG is 2% of the total soil mass.

#### Effect of XG

**Fig. 12:**
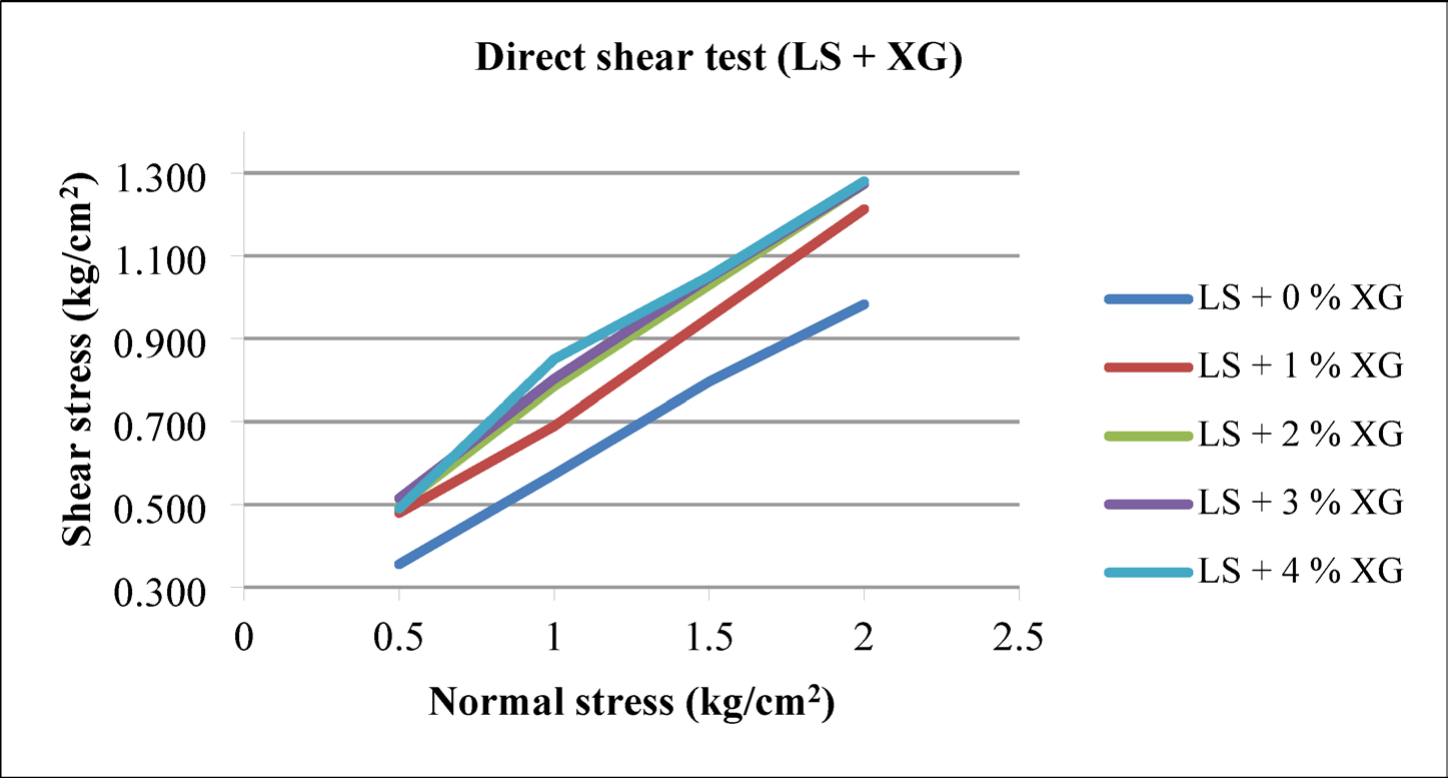
Failure envelopes of laterite soil – XG mixes.

The friction angle increases slightly as the XG content increases compared with laterite soil. Conversely, the cohesion intercept increases in correlation with the XG content, with a notable increment in cohesion observed from 0.15 kg/cm^2^ to 0.279 kg/cm^2^ at the 3% optimum XG dosage. This represents an approximately 86% augmentation in cohesion compared to traditional laterite soil. Figure 12 depicts failure envelopes of all the laterite soil – XG biopolymer mixes. The angle of internal friction (φ) and cohesion intercept (c) of the mixes were obtained from linear regression analyses.

#### Effect of GG

**Fig. 13:**
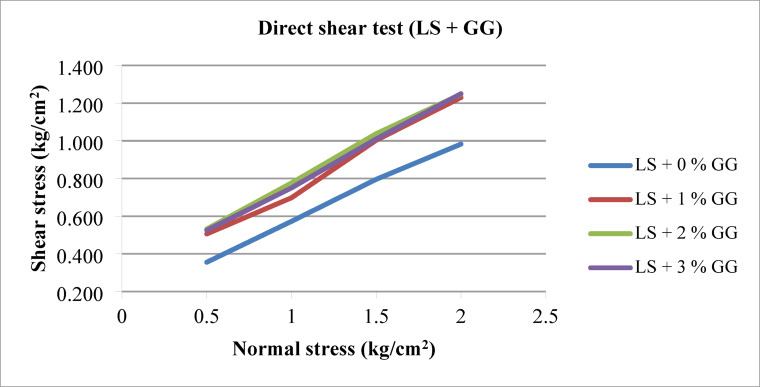
Failure envelopes of laterite soil – GG mixes.

Figure 13 shows failure envelopes of all the laterite soil – GG biopolymer mixes. The angle of internal friction (φ) and cohesion intercept (c) of the mixes were obtained from linear regression analyses. The friction angle increases as the GG content increases compared to laterite soil alone. Conversely, the cohesion intercept rises in correlation with the GG content, with a notable increase in cohesion observed from 0.15 kg/cm^2^ to 0.33 kg/cm^2^ at the 2% GG dosage. This represents an approximately 120% increase in cohesion compared to traditional laterite soil. The overall shear strength also appears to increase, as indicated by the shear stresses at the point of failure. No such change is observed in angle of shearing resistance as GG varies from 1% to 3% in laterite soil. The carbon component in GG can act as a binder, which helps stabilize the soil particles by increasing the cohesion between them.

### CBR test

#### Effect of XG

**Fig. 14:**
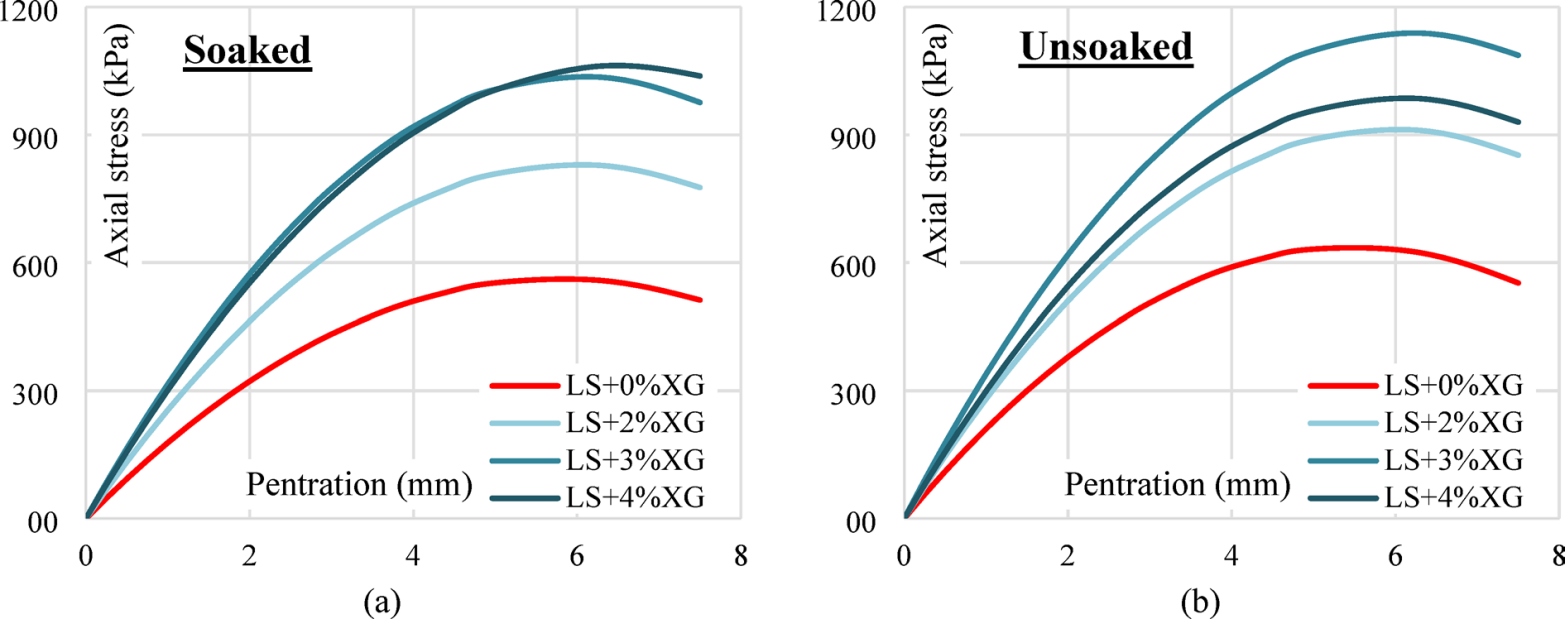
(**a**) Soaked condition CBR curves of untreated and XG treated laterite soil (**b**) Unsoaked condition CBR curves of untreated XG treated laterite soil.

In the unsoaked condition, the CBR value of untreated soil (LS + 0%XG) is 8.37%. With the addition of 1% XG, the CBR slightly increases to 8.67%, followed by a significant rise at 2% XG (10.19%) and peaking at 3% XG with a value of 12.57%. However, a slight reduction is observed at 4% XG (11.67%), indicating that 3% XG may be the optimal dosage for maximum strength improvement under unsoaked conditions. Similarly, in the soaked condition, the CBR increases steadily from 7.07% (untreated) to 11.49% at 3% XG, then slightly decreases to 11.01% at 4% XG. This trend again suggests that the most effective dosage of XG is around 3%, beyond which the improvement starts to taper off. Figure 14a,b illustrates the CBR curves for both untreated and XG-treated samples in soaked and unsoaked condition respectively.

**Fig. 15:**
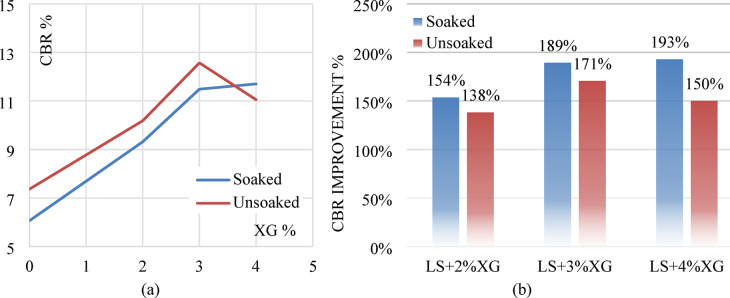
(**a**) Soaked and unsoaked CBR of laterite soil with varying XG contents (**b**) CBR % improvement of laterite soil with varying XG contents.

Figure 15a shows the variation of CBR values with different percentages of XG for both soaked and unsoaked conditions. A consistent increase in CBR is observed with the addition of XG up to 3%, beyond which a slight reduction occurs at 4%. The improvement is more pronounced in the unsoaked condition compared to the soaked state, although both follow a similar trend. The slight reduction at 4% XG may be due to excessive biopolymer coating around soil particles, which introduces lubrication effects and reduces frictional resistance. Figure 15b presents the percentage improvement in CBR for different XG contents compared with untreated laterite soil. Laterite soil with 3% XG showed a 171% increase in unsoaked CBR value and a 189% increase in soaked CBR value. The overall enhancement in both soaked and unsoaked CBR values with increasing XG content can be attributed to the binding and stabilizing effect of the XG. It likely fills the soil pores and improves particle cohesion, leading to increased resistance against penetration. The slight drop in CBR at 4% may be due to oversaturation of the polymer, leading to reduced stiffness or excessive water absorption.

#### Effect of GG

**Fig. 16:**
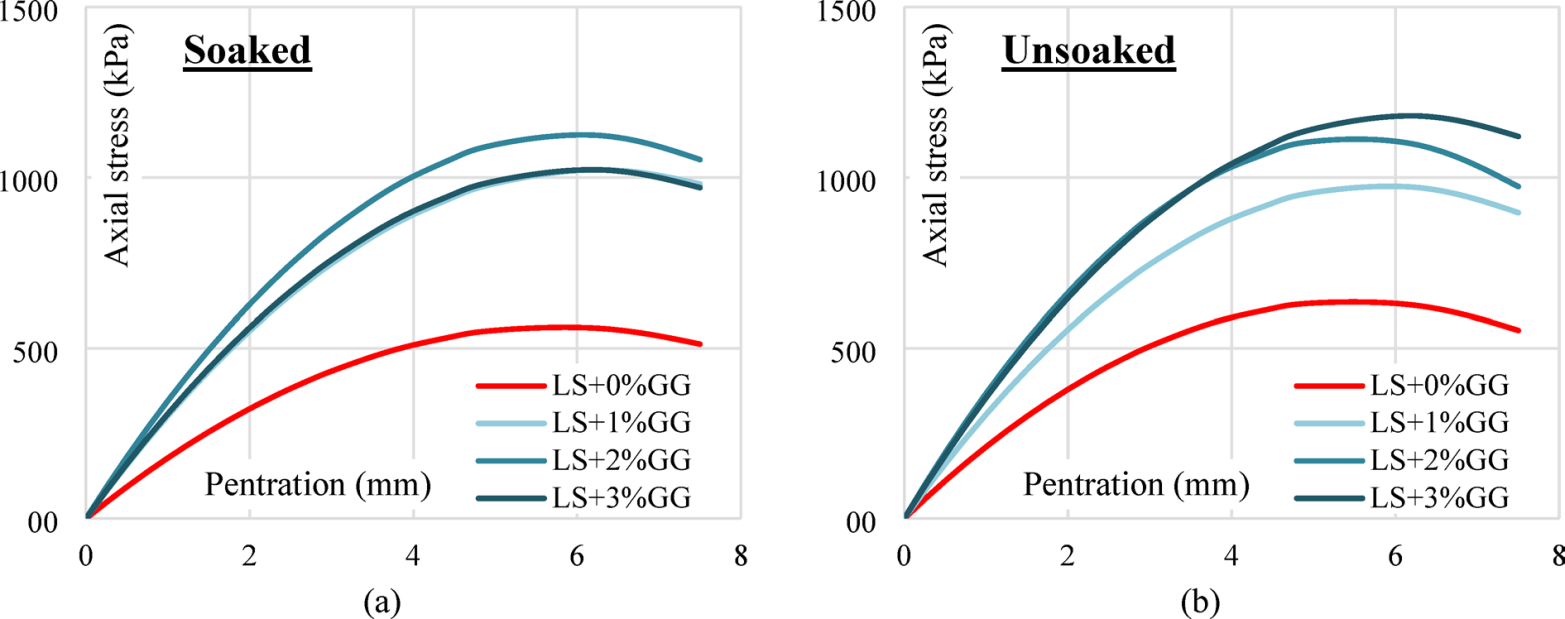
(**a**) Soaked condition CBR curves of untreated and GG treated laterite soil (**b**) Unsoaked condition CBR curves of untreated GG treated laterite soil.

In the unsoaked condition, the untreated laterite soil (LS + 0%GG) shows a CBR value of 8.37%. Upon adding 1% GG, the value increases to 10.62%, followed by a further increase to 13.22% at 2% GG content, indicating enhanced strength and load-bearing capacity. However, at 3% GG, the CBR slightly drops to 11.70%, suggesting a decline in performance beyond the optimum dosage. A similar trend is observed under the soaked condition. The CBR value rises from 7.07% (0% GG) to 9.75% with 1% GG, reaching a peak of 12.57% at 2% GG. At 3% GG, the value decreases to 10.62%, confirming that 2% GG is likely the most effective concentration for improving CBR under soaked conditions. Figure 16a,b illustrates the CBR curves for both untreated and GG-treated samples in soaked and unsoaked condition respectively.

**Fig. 17:**
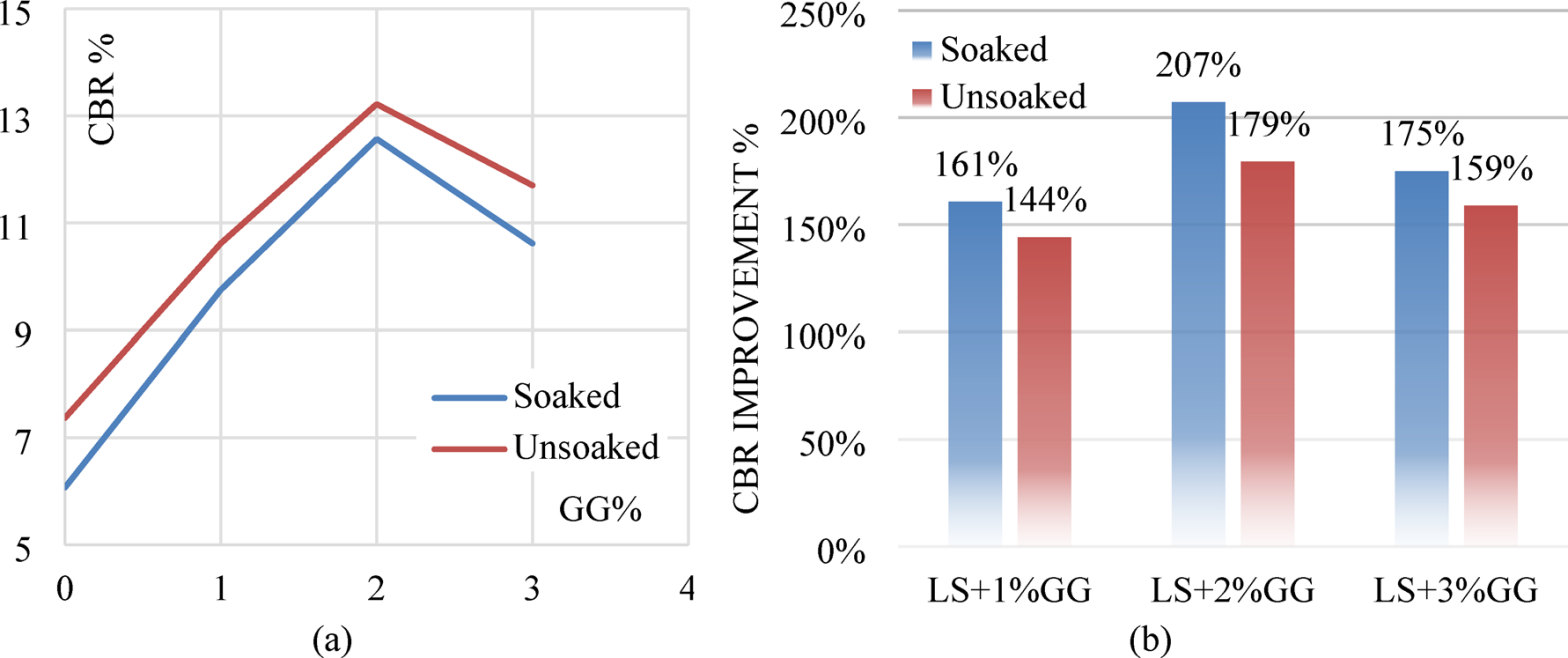
(**a**) Soaked and unsoaked CBR of laterite soil with varying GG contents (**b**) CBR % improvement of laterite soil with varying GG contents.

The improvement in CBR values with increasing GG content up to 2% can be attributed to the biopolymer’s ability to bind soil particles, reduce void spaces, and increase cohesion, thus enhancing the overall stiffness and resistance to penetration. Laterite soil with 2% XG showed a 179% increase in unsoaked CBR value and a 207% increase in soaked CBR value. The slight decline in CBR at 3% GG may result from excessive gum content, which can lead to the formation of thick films around soil particles, reducing inter-particle friction or causing brittleness upon drying. Figure 17a shows the soaked and unsoaked CBR values with different GG contents and Fig. 17b depicts the % improvement in CBR values in both conditions.

### Falling head permeability test

**Fig. 18:**
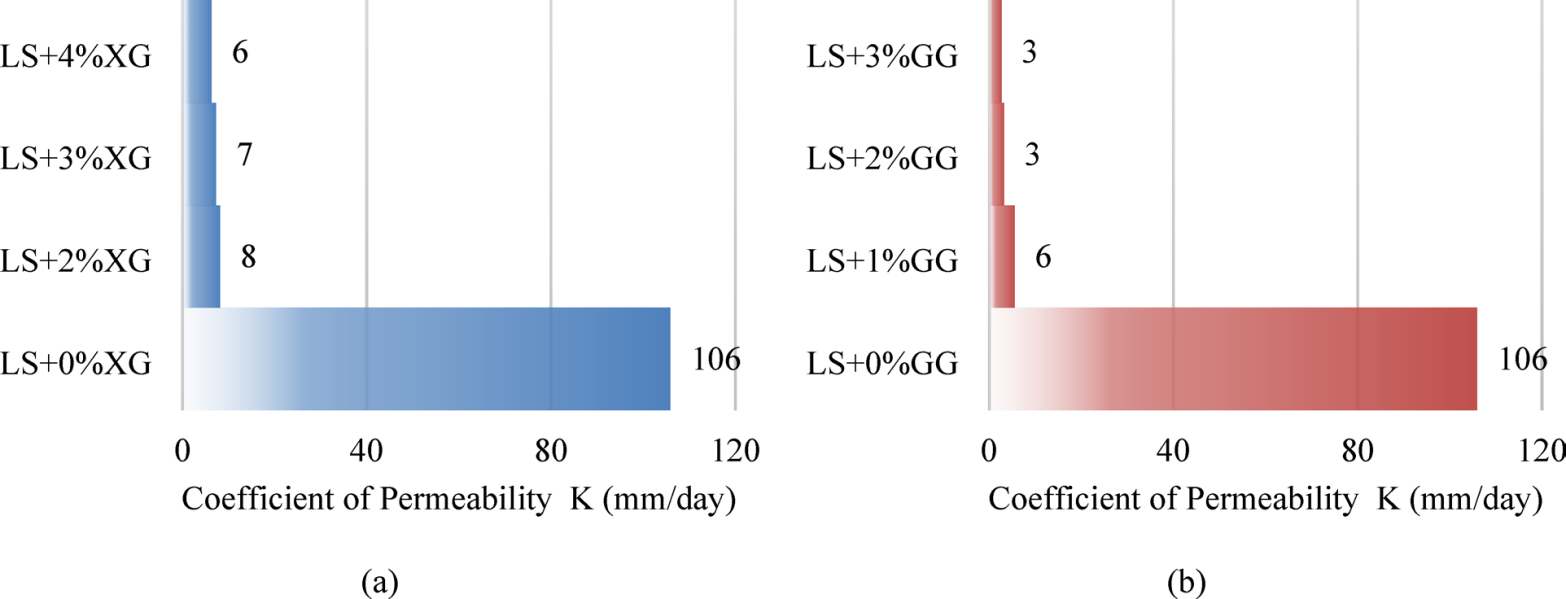
The results of falling head test for laterite soil treated with (**a**) XG (**b**) GG.

The results of the falling head test for laterite soil treated with XG and GG are shown in Fig. 18a,b, highlighting the impact of biopolymers on permeability. The coefficient of permeability was determined using a falling head permeability test (IS:2720 Part 17) conducted on compacted specimens prepared at the optimum moisture content and maximum dry density. The inclusion of XG biopolymer results in a decrease in soil permeability. The permeability coefficient of regular laterite soil measured 1.22555 × 10^− 6^; however, at the ideal concentration of 3%, it decreased to 8.42708 × 10^− 8^. The permeability falls by around 93.12% at the optimum dose of 3%. As the amount of XG in the soil matrix improves, cross-linking components fill the pores and reduce the coefficient of permeability, preventing water from passing through the sample. For laterite soil, LS + 1%XG, LS + 2%XG, LS + 3%XG and LS + 4%XG mixtures, the permeability coefficients were 122.55 × 10^− 8^, 16.808 × 10^− 8^, 9.387 × 10^− 8^, 8.42 × 10^− 8^ and 7.29 × 10^− 8^ respectively. Adding GG biopolymer reduces soil permeability, but at the optimum dosage of 2%, it diminished to 3.7204 × 10^− 8^. GG accumulates in soil gaps to produce hydrogel and link soil particles, limiting permeability. The permeability falls by around 96.96% at the optimum dose of 2%. For laterite soil, LS + 1%GG, LS + 2%GG, and LS + 3%GG mixtures, the permeability coefficients were 122.55 × 10^− 8^, 6.422 × 10^− 8^, 3.720 × 10^− 8^, and 3.064 × 10^− 8^, respectively.

**Fig. 19:**
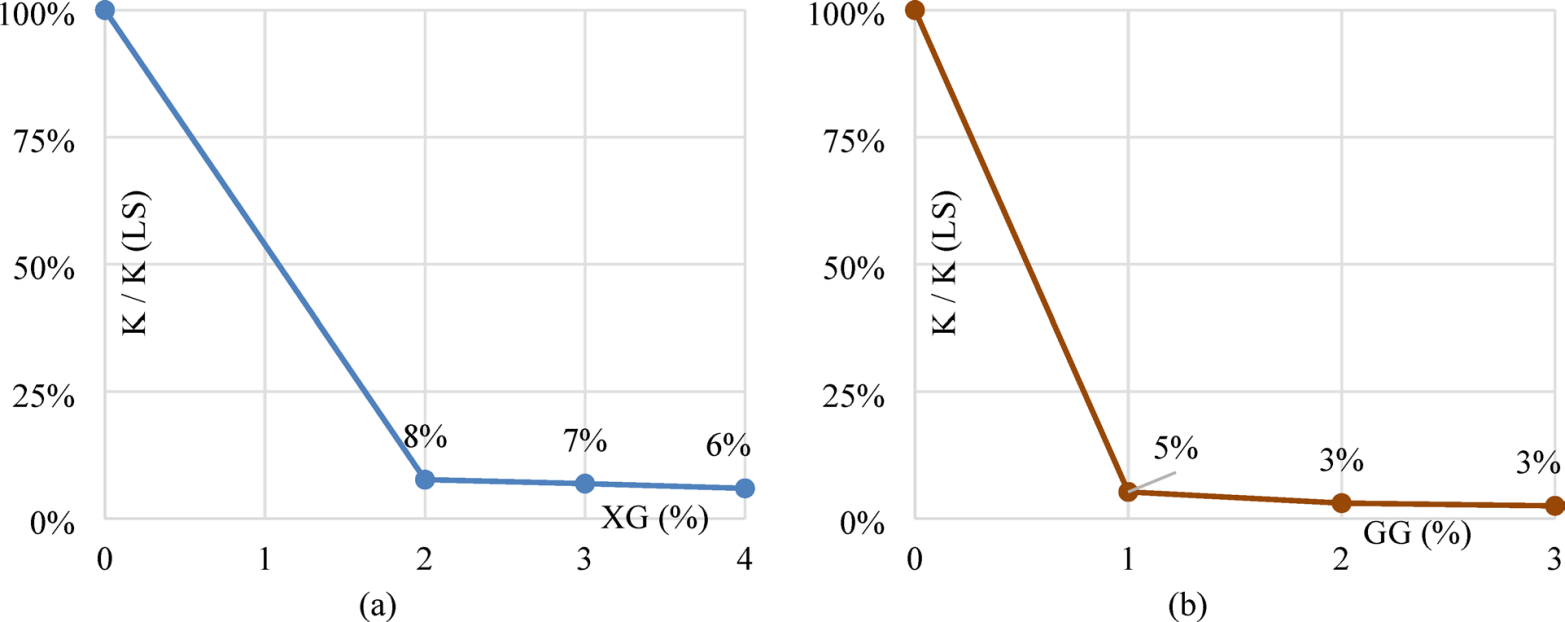
Permeability coefficients for laterite soil with (**a**) XG biopolymer mixes (**b**) GG biopolymer mixes.

Figure 19a shows the permeability ratio K(LS + XG)/K(LS) decreases steadily with increasing XG content, falling from 100% at 0% XG (baseline) to about 8% at 2% XG, 7% at 3% XG and 6% at 4% XG. The gradual, almost linear decline shows that XG progressively restricts water flow as more biopolymer is added. Figure 19b shows the permeability ratio K(LS + GG)/K(LS) drops extremely sharply at GG dosages — from 100% at 0% to ~ 5% at 1% GG, 3% at 2% GG and 2.4% at 3% GG.

### Odometer test

#### Effect of XG

**Fig. 20:**
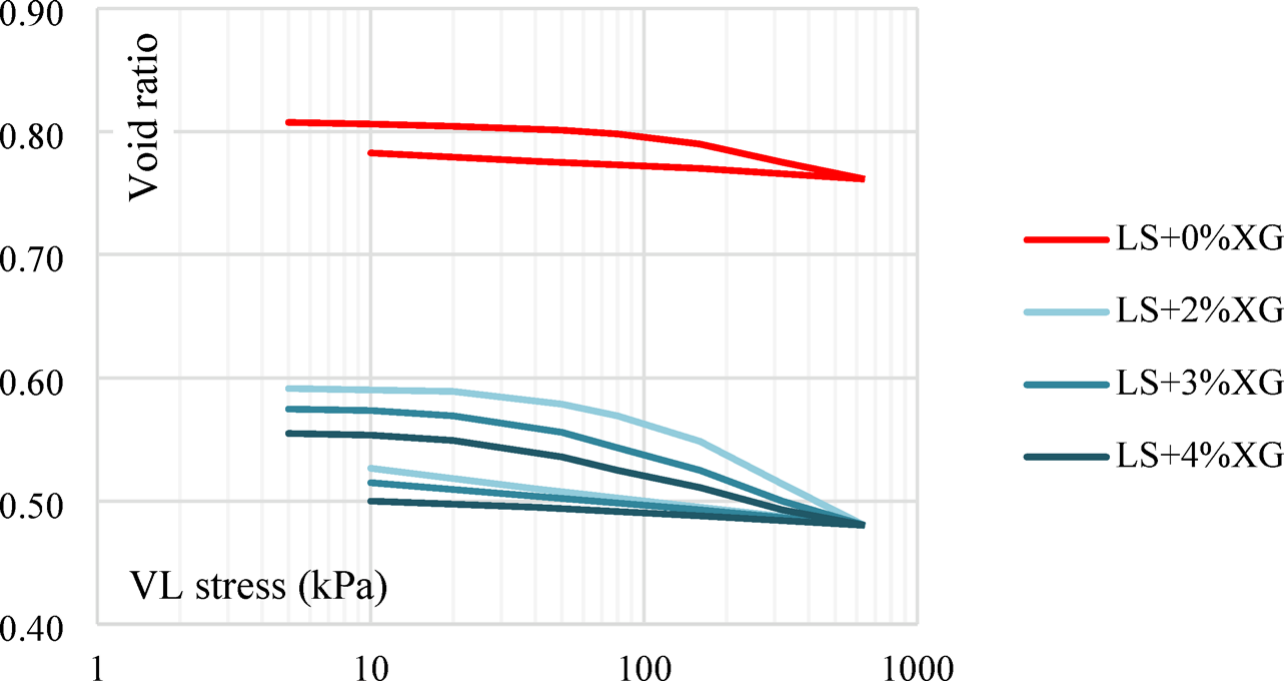
One-dimensional consolidation test results for untreated and treated laterite soil with XG.

Untreated laterite soil’s compression curve was significantly higher than the treated XG curves. The compression characteristics of untreated laterite soil were found to exhibit significantly higher values when compared to the specimens treated with XG. This observation highlights the beneficial impact of XG treatment on the soil’s compressibility. In particular, when subjected to consolidation tests, the untreated laterite soil exhibited a relatively high initial void ratio of 0.807. However, upon treatment with an optimum dose of XG, this void ratio significantly decreased to 0.4803. This decrease in the void ratio signifies a noteworthy consolidation process within the soil. It suggests that XG treatment reduces soil compressibility and boosts load-bearing capacity, making it a promising method for enhancing laterite soil engineering qualities in building, roadwork, and other civil engineering applications. The one-dimensional consolidation test results compare untreated laterite soil with samples treated using XG biopolymer shown in Fig. 20.

**Fig. 21:**
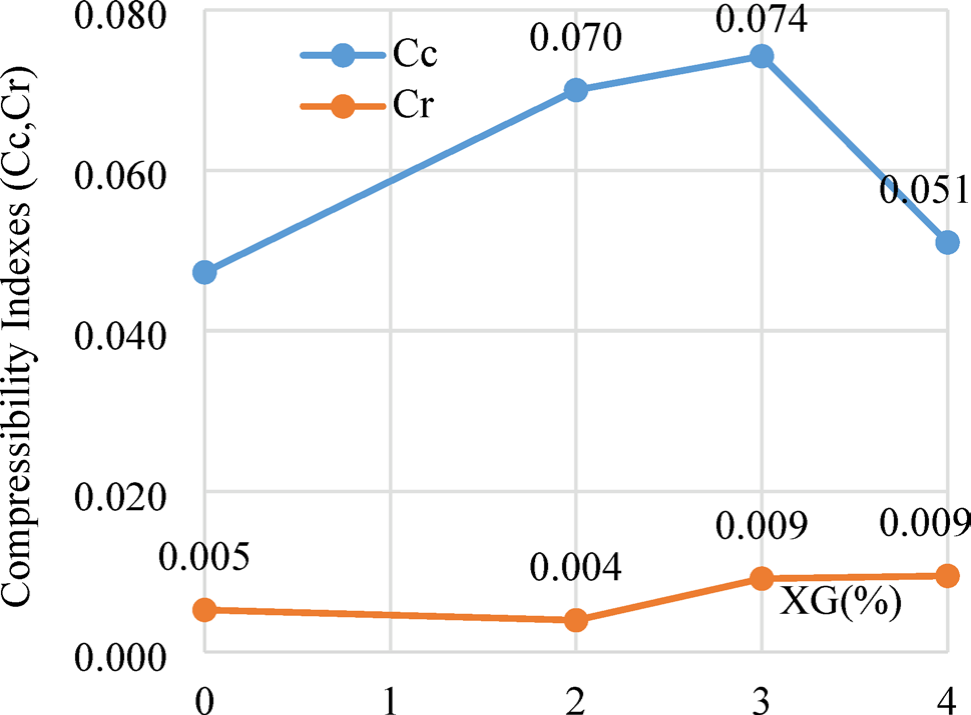
Compressibility indexes for untreated and treated laterite soil with XG.

Figure 21 illustrates the variation of the compression index (Cc) and recompression index (Cr) with different XG contents. The Cc initially increases with XG addition, attaining a maximum at 3% XG, followed by a slight decline at higher dosages. Conversely, the Cr exhibits a steady rise up to 3% XG, suggesting improved elastic response during the recompression phase. The observed trend up to 3% indicates an enhanced ability for controlled deformation during primary consolidation, likely resulting from hydrogel formation and the uniform dispersion of XG within the soil structure.

#### Effect of GG

**Fig. 22:**
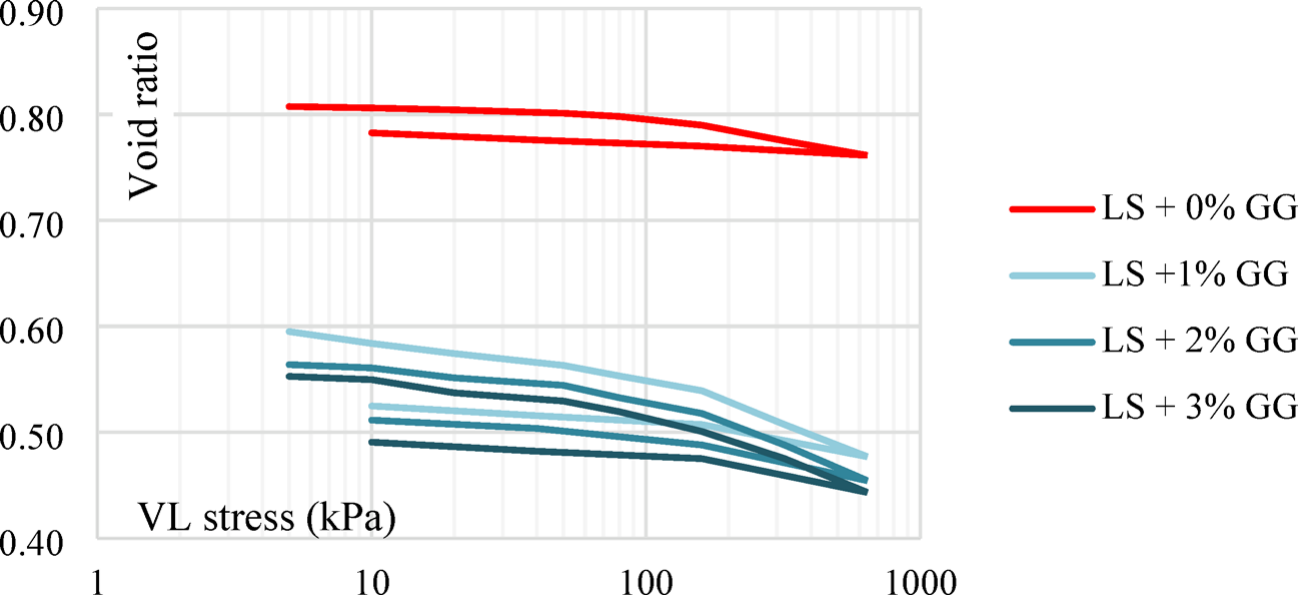
One-dimensional consolidation test results for untreated and treated laterite soil with GG.

The presence of voids or air gaps within the laterite soil sample tends to decrease when an optimal dosage of GG is added. This is evident from the reduction in the void ratio from 0.807 to 0.454. Such a significant decrease in the void ratio suggests that the soil structure becomes denser and more compact. This densification is typically associated with soil compression or consolidation, processes that occur when the soil particles are rearranged due to the influence of external loads or binding effects of the biopolymer. The one-dimensional consolidation test results compare untreated laterite soil with samples treated using GG biopolymer shown in Fig. 22.

**Fig. 23:**
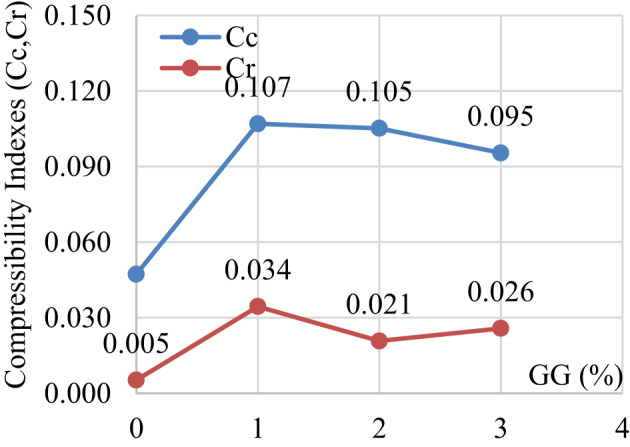
Compressibility indexes for untreated and treated laterite soil with GG.

Figure 23 illustrates the variation of the compression index (Cc) and recompression index (Cr) with the addition of GG. The Cc increases sharply with GG addition up to 1%, after which it slightly decreases. The initial increase in Cc can be attributed to flocculation and aggregation of soil particles caused by the interaction of GG with the minerals, which increases compressibility. The recompression index (Cr) also increases with GG addition, with a relatively stable trend between 2% and 3% GG.

### XRD test

#### Effect of XG

**Fig. 24:**
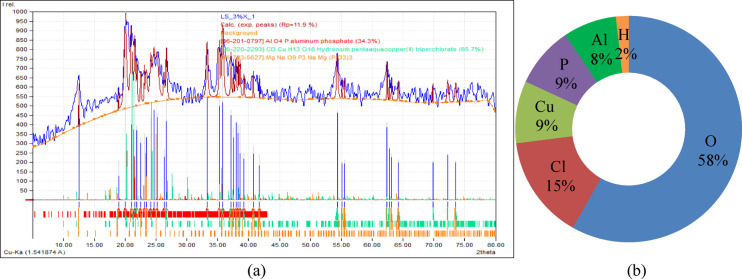
(**a**) XRD pattern (**b**) Elemental composition analysis of LS + 3%XG soil mix.

An XRD analysis was conducted on laterite soil with an ideal dosage of 3% XG. The final composition of the XG biopolymer blended with laterite soil consists of 58.2% oxygen, 14.8% chlorine, 8.9% copper, 8.7% phosphorus, 7.6% aluminum, and 1.8% hydrogen. Soil is made more cohesive and binding by adding XG. The soil is stabilized by xanthan gum because it absorbs water. Minimizing shrinkage and cracking, it promotes soil moisture. The cohesive and binding properties of XG protect soil from erosion caused by wind and water. Environmentally conscious people should use XG. Conventional chemical additives may adversely affect ecosystems and contaminate groundwater; however, this eco-friendly soil stabilization approach helps reduce such environmental risks. Figure 24a presents the XRD results and 24(b) shows the elemental composition of untreated laterite soil and the soil mixture containing 3% XG.

#### Effect of GG

**Fig. 25:**
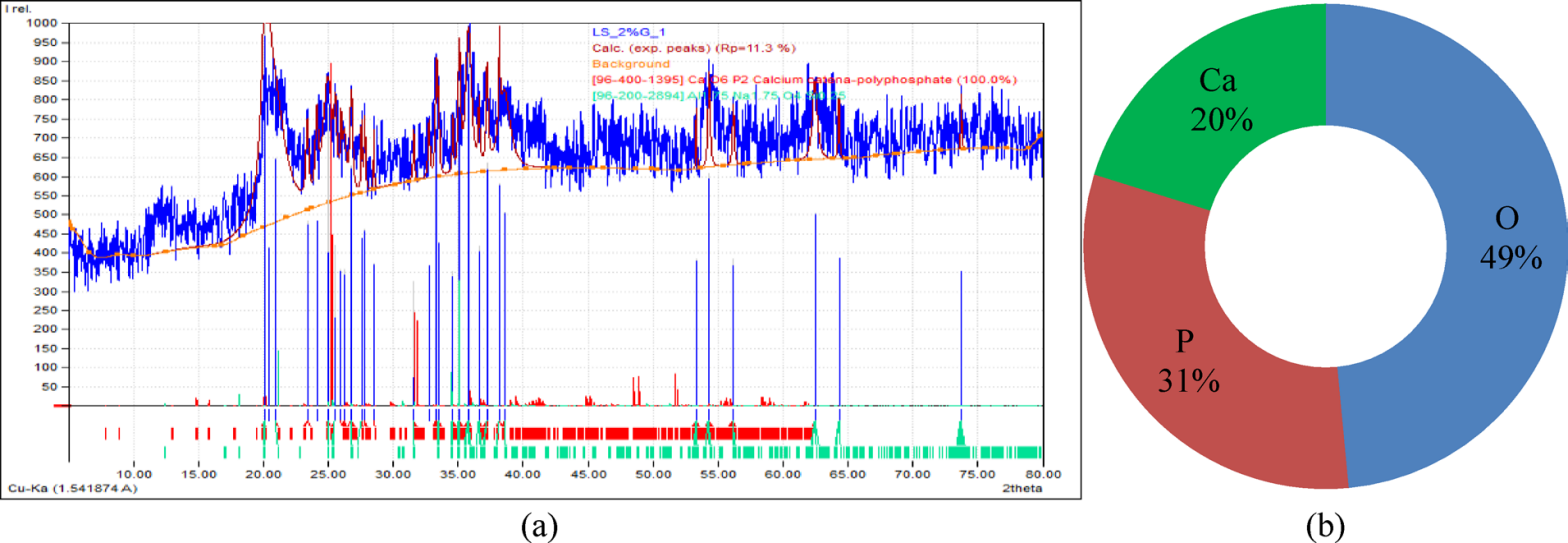
(**a**) XRD pattern (**b**) Elemental composition analysis of LS + 2%GG soil mix.

XRD study of laterite soil and 2% GG (optimum dose) was done. The blend had 48.50% oxygen, 31.30% phosphorus, and 20.20% calcium. Adding GG biopolymer to laterite soil increased its engineering qualities. GG increases soil particle cohesiveness, improving structure and reducing erosion. This is useful on slopes and building sites that erode. GG improves soil porosity, permeability, and compaction. Stabilizing loose or sandy soils makes them better for construction. Figure 25a show laterite soil and 2% GG soil mix XRD results and Fig. 25b Its elemental composition.

### Scanning electron microscopy (SEM)

#### Effect of XG

**Fig. 26:**
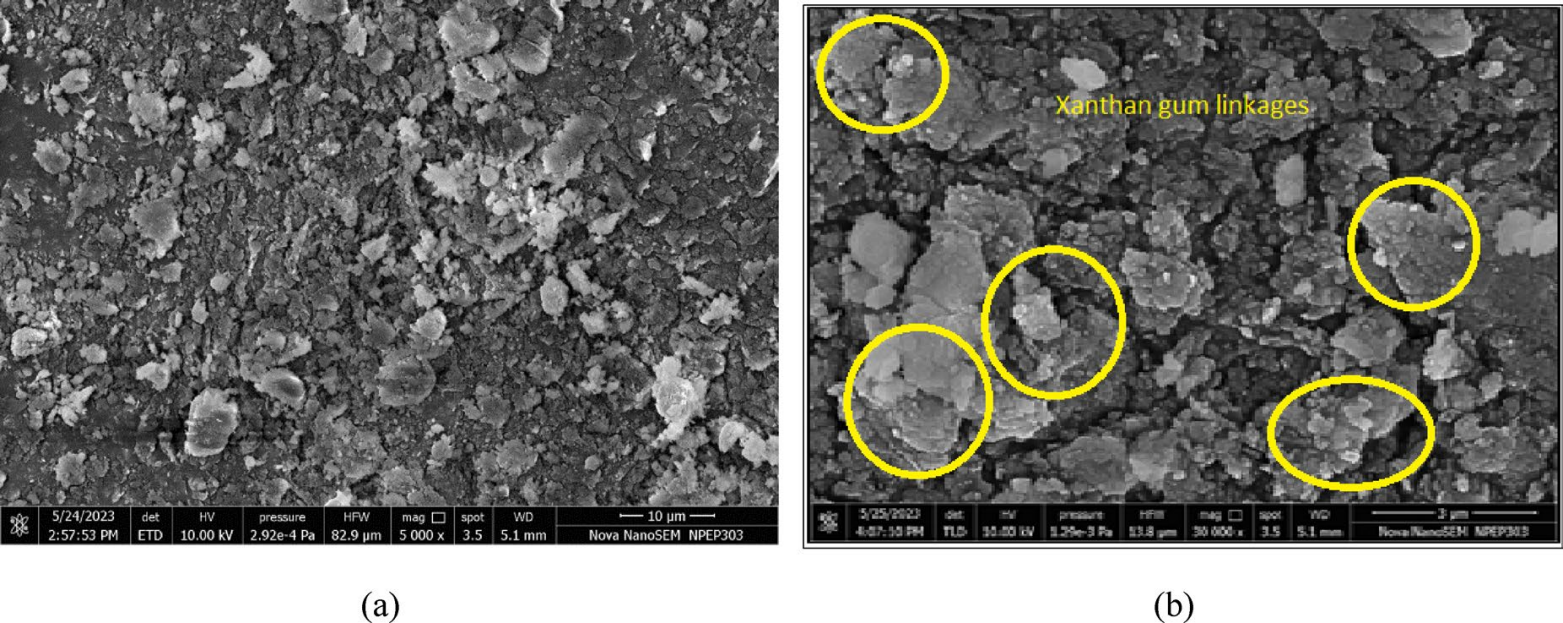
SEM micrographs of (**a**) Virgin laterite soil (**b**) LS + 3% XG soil mix.

Figure 26a reveals that the untreated laterite soil surface is irregular, with poorly bonded particles and a rough texture. The microstructural and mineralogical characterization of laterite soil treated with 3% XG provides significant insights into its stabilization mechanism. The SEM observations further confirmed the development of cementitious compounds within the XG–laterite soil matrix (Fig. 26b , marked regions). These products effectively hydrogen bonded soil particles, filled voids, and modified surface morphology, contributing to a denser and more stable structure. The micrographs also demonstrated direct physicochemical interactions between XG and fine soil particles due to electrostatic attraction, leading to polymer cation bridge formation that increased interparticle contact and cohesion. As a result, XG not only improves water retention and reduces shrinkage and cracking but also enhances the resistance of soil to erosion, offering an environmentally sustainable approach to soil stabilization.

#### Effect of GG

**Fig. 27:**
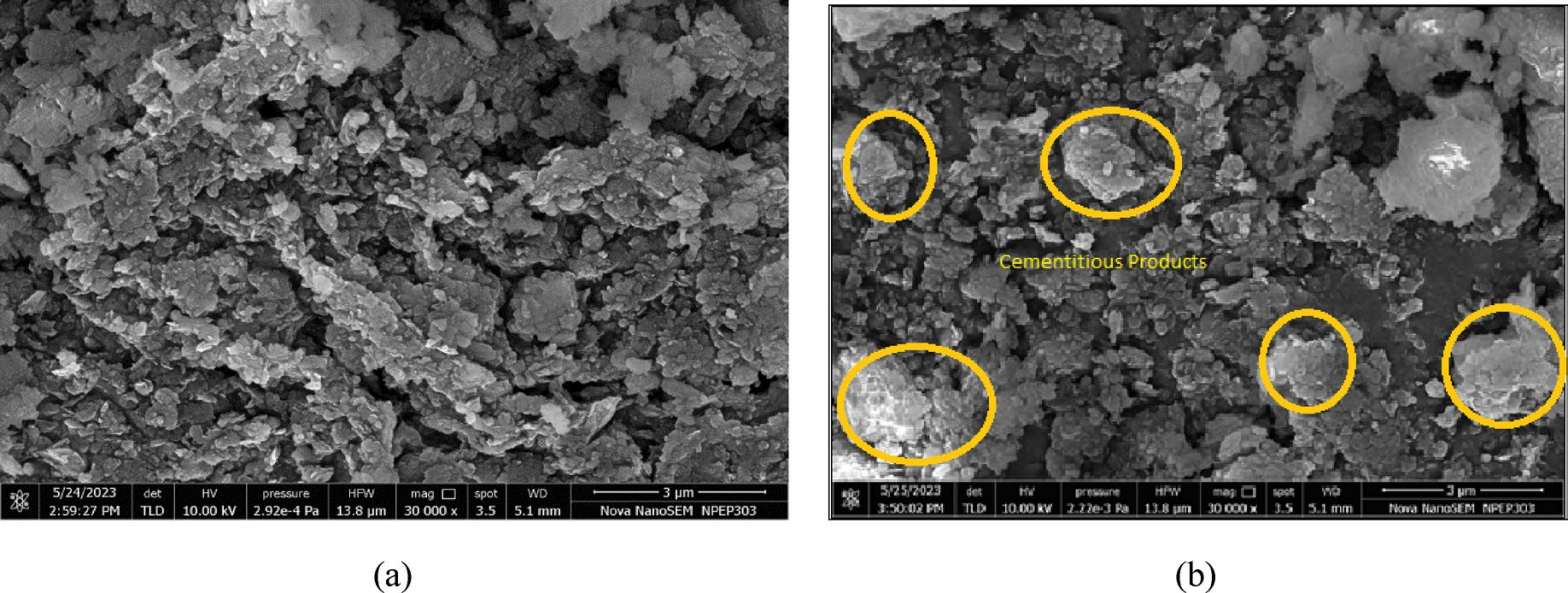
SEM micrographs of (**a**) Virgin laterite soil (**b**) LS + 2% GG soil mix.

SEM analysis was carried out to better understand how GG improves the shear strength of laterite soil. The images show that GG fills the gaps between soil particles, forming hydrogels that help bind the particles together. The strength of the treated soil depends on how strong and dense these hydrogel bonds are inside the pores. The formation of hydrogels also changes the structure of the soil matrix, making it more stable and cohesive. Figure 27a shows the SEM images of virgin laterite soil and 27(b) shows the laterite soil mixed with 2% GG, where the cementitious hydrogel products can be seen in the marked areas.

## Conclusions

This study examined the potential of biopolymers xanthan gum (XG) and guar gum (GG) for laterite soil stabilization. The research showed that treated soils improved in strength and geotechnical behavior through UCS, direct shear test, permeability, void ratio, and microstructural examination. The findings demonstrate biopolymer stabilization’s environmental and technological efficacy in improving problematic soils.

- The addition of biopolymers significantly improved the unconfined compressive strength (UCS) of laterite soil. A maximum UCS of 443.56 kPa was achieved with 3% XG after 28 days, marking a 301.40% increase compared to untreated soil. For 2% GG, UCS reached 332.67 kPa, representing a 201.05% increase after 28 days. These results confirm that both biopolymers are highly effective in increasing the load-bearing capacity of laterite soil.
- The addition of biopolymers significantly enhanced the cohesion of laterite soil in proportion to dosage. At 3%, XG increased cohesion from 0.15 to 0.279 kg/cm² (86% increase), while GG increased it to 0.33 kg/cm² (120% increase), demonstrating the effectiveness of both biopolymers for sustainable soil stabilization.
- The permeability of laterite soil decreased substantially with biopolymer addition. A 3% dosage of XG reduced permeability by approximately 93.12%, while a 2% dosage of GG achieved a 96.96% reduction.
- The void ratio decreased from 0.807 (untreated) to 0.4803 for 3% XG and to 0.454 for 2% GG, indicating better compactness and consolidation behavior in treated soils.
- The study determined that 3% XG and 2% GG are the optimum dosages for maximum enhancement in strength and durability of laterite soil, balancing performance and material economy.
- Cementitious compounds were found and improved particle bonding was verified by XRD investigations, which led to increased strength and durability.
- Xanthan and guar gum are biodegradable and sustainable alternatives to conventional stabilizers, making them suitable for environmentally conscious geotechnical applications.

For researchers, these results provide guidance on the relationship between biopolymer content and enhancements in cohesion, compressive strength, and compressibility of laterite soil. For practicing engineers, the study establishes practical performance benchmarks, such as significant improvements in unconfined compressive strength and reductions in permeability, indicating that XG and GG treatments are suitable for pavement subgrades, embankments, and geo-environmental barriers.

Future research must focus on large-scale field experiments and long-term monitoring to assess the real-world performance of biopolymer-treated soils. Furthermore, investigating the combined impacts of biopolymers and other sustainable additives may improve soil stability and expand their applicability in a variety of geotechnical engineering environments.

## Data Availability

The datasets generated and/or analysed during the current study are available from the corresponding author upon reasonable request.
